# Molecular and Biological Characterization of a New Strawberry Cytorhabdovirus

**DOI:** 10.3390/v11110982

**Published:** 2019-10-24

**Authors:** Jana Fránová, Jaroslava Přibylová, Igor Koloniuk

**Affiliations:** Department of Plant Virology, Institute of Plant Molecular Biology, Biology Centre, Czech Academy of Sciences, 370 05 České Budějovice, Czech Republic; pribyl@umbr.cas.cz

**Keywords:** novel cytorhabdovirus, high-throughput sequencing, aphid transmission, mechanical inoculation, grafting-mediated virus transmission, *Fragaria* spp., *Chaetosiphon fragaefolii*

## Abstract

Virus diseases of strawberry present several complex problems. More than 25 viruses have been described in the genus *Fragaria* thus far. Here, we describe a novel rhabdovirus, tentatively named strawberry virus 1 (StrV-1), that infects *F. ananassa* and *F. vesca* plants. Genomic sequences of three distinct StrV-1 genotypes co-infecting a single *F. ananassa* host were obtained using combined Illumina and Ion Proton high-throughput sequencing. StrV-1 was transmitted to herbaceous plants via *Aphis fabae* and *A. ruborum*, further mechanically transmitted to *Nicotiana occidentalis* 37B and sub-inoculated to *N. benthamiana*, *N. benthamiana* DCL2/4i, *N.* *occidentalis* 37B, and *Physalis floridana* plants. Irregular chlorotic sectors on leaf blades and the multiplication of calyx leaves seem to be the diagnostic symptoms for StrV-1 on indexed *F. vesca* clones. StrV-1 was detected in asymptomatic grafted plants and in 49 out of 159 field strawberry samples via RT-PCR followed by Sanger sequencing. The bacilliform shape of the virions, which have a cytoplasm-limited distribution, their size, and phylogenetic relationships support the assignment of StrV-1 to a distinct species of the genus *Cytorhabdovirus*. *Acyrthosiphon malvae*, *A. fabae*, and *A. ruborum* were shown to transmit StrV-1 under experimental conditions.

## 1. Introduction

Strawberry, as a very popular commercial and garden fruit and nutritionally important fruit, has long been cultivated worldwide [1]. Strawberries are grown in many countries in annual plasticulture systems. However, based on the conditions of the regional agriculture practices, the triennial/quadrennial matted-row production system is used in the Czech Republic as well. Therefore, great emphasis is placed on ensuring the health of the propagation material and on plant protection during growing seasons [2].

Viruses in strawberry plants are found at low concentrations and in mixed infections, and commonly induce non-specific plant symptoms [3,4]. In particular, multiple viral infections in plants can lead to yield loss and plant decline [2,3,5,6,7,8,9,10,11]. A recent study showed that the number and weight of fruits from strawberry plants with an asymptomatic infection with only strawberry mild yellow edge virus (SMYEV, family: *Alphaflexiviridae*, genus: *Potexvirus*) was reduced by 28% to 63% compared with that of healthy plants, depending on the parameter measured and the production cycle [12,13].

More than 25 virus and virus-like agents have been reported in strawberry [2]. Classical molecular-biological methods (indicator clone grafting, virus transmission by aphids, PCR) have indicated that the most important strawberry viruses are SMYEV, strawberry crinkle virus (SCV, *Rhabdoviridae*, *Cytorhabdovirus*), strawberry mottle virus (SMoV, *Secoviridae*), and strawberry vein banding virus (SVBV, *Caulimoviridae*, *Caulimovirus*), especially when occurring in mixed infections [3,9,11]. Additionally, a new accession was characterized as strawberry chlorotic fleck-associated virus (SCFaV, *Closteroviridae*, *Closterovirus*) [14]. *F. ananassa* cultivated in China was found to be a new host for cucumber mosaic virus [15]. However, after the introduction of high-throughput sequencing (HTS), the number of novel viruses that have been identified has increased. Strawberry polerovirus-1 (SPV-1, *Luteoviridae*, *Polerovirus*) was first described in eastern Canada in plants showing decline symptoms [11] and was subsequently detected in the USA [16] and in Argentina [17]. Strawberry crinivirus 4 (SCrV-4, *Closteroviridae*, *Crinivirus*) was first reported in Canada [18], and nucleotide sequences of new strawberry crinivirus 3 (SCrV-3) have been identified in the USA and China [19].

In addition to the vegetative propagation of systemically infected plant materials in stock nurseries, strawberry viruses are transmitted by insects, nematodes, and other vectors. Aphids are the most significant vectors. Among aphids, *Chaetosiphon fragaefolii* (Cockerell, 1901) (Homoptera: Aphididae) has been shown to be an effective vector of strawberry viruses in the field. However, other species of aphids have also been shown to effectively infect strawberry [3].

The family *Rhabdoviridae* (order *Mononegavirales*) consists of 18 genera characterized by viruses with single-stranded negative-sense RNA genomes and contains 135 recognized species [20]. Rhabdoviruses infect a wide range of host species, including vertebrates, invertebrates, and plants. Most are transmitted by insect vectors to their vertebrate or plant hosts. Four genera of the *Rhabdoviridae* family encompass plant-infecting members. One genus, *Cytorhabdovirus*, includes viruses with non-segmented genomes that replicate in the cytoplasm of infected cells [21]. Over 90 putative plant rhabdoviruses have been identified based on the symptomatology of host species and distinct rhabdoviral particle morphologies. Due to a lack of molecular descriptions, many rhabdoviruses have a provisional taxonomic status [21,22,23,24,25]. Five canonical protein-coding genes of plant-specific rhabdoviruses may be supplemented by various numbers of accessory genes [26].

The complete nucleotide sequences of more than 30 plant-infecting rhabdoviruses are available. Our knowledge of rhabdoviruses infecting strawberry plants is limited. SCV occurs worldwide and is one of the most harmful strawberry viruses. Strawberry latent C virus (SLCV) was reported in the United States, Nova Scotia (Canada) and Japan [9]. Particles of SLCV accumulate in the perinuclear spaces of infected cells, in contrast to those of SCV, which accumulate in the cytoplasm [27]. Additional knowledge of SLCV is limited, and an SLCV genomic sequence is currently lacking.

Recently, we reported on the complete sequence of two SCV isolates infecting a single strawberry plant [28]. In addition to SCV, several contigs of other cytorhabdoviruses unrelated to SCV have been identified. Here, we characterized the virus tentatively named strawberry virus 1 (StrV-1), which was found to infect garden and wild strawberry plants.

## 2. Materials and Methods

### 2.1. Strawberry Samples

For HTS, three strawberry plants showing symptoms resembling those of viral infection were selected: (i) *F. ananassa* Duch. cv. Čačanská raná (the ČRM1 isolate) was imported to the Research and Breeding Institute of Pomology (RBIP, Holovousy, Czech Republic) from former Yugoslavia in 1989. The daughter plant was cultivated from stolon kindly provided by Mrs. M. Erbenová in 1993 and has been maintained in the Institute of Plant Molecular Biology (IPMB, Biology Centre of the CAS) to this day [28]. (ii) A plant of *F. vesca semperflorens* cv. Rujana (the 1/2017 isolate) was cultivated from commercially available seeds at a private garden in South Bohemia (locality Třísov, Czech Republic) from spring 2016. In April 2017, the plant was moved to IPMB in České Budějovice. (iii) *F. ananassa* cv. Elkat (the 9/2018 isolate) with irregular vein clearing and necroses originated from a strawberry production field in South Bohemia.

During 2016–2019, strawberry plants showing growth abnormalities were additionally sampled at several locations within the Czech Republic (Table S1). The above-mentioned plants, which were used for HTS, and 63 strawberry plants selected from strawberry screening were individually planted in pots and transferred and maintained at the open experimental field in the IPMB.

Seeds of *F. vesca* ‘Alpine’ were kindly provided by Mrs. M. Erbenová in 1993. Mother plants of *F. vesca* indicator clones EMC, EMK, FV-72, and UC-6 (negative for SMoV, SCV, SMYEV, SPV-1, StrV-1, and SVBV—personal communication) were kindly provided by Mrs. L. Valentová and Dr. R. Čmejla in 2019, both from RBIP Holovousy. Daughter plants used for grafting were cultivated in an insect-proof greenhouse at IPMB. Leaves from seedlings of *F. vesca* ‘Alpine’ were used as healthy controls for molecular analyses.

### 2.2. Aphid Screening and Identification

Strawberry plants growing in the experimental field in IPMB and in a few private gardens in South Bohemia were screened for aphid presence from 2016 to 2019. Aphids were kindly identified by Dr. J. Havelka (Biol Ctr., Czech Acad Sci) according to their morphological characteristics. Molecular identification was conducted using direct PCR from a single aphid with primers specific to either the cytochrome c oxidase subunit 1 (*COI*) [29] or cytochrome B (*cytb*) [30] gene (Table S2) and with Phire Plant Direct PCR Master Mix (Thermo Fisher Scientific, Vilnius, Lithuania). The PCR products were either directly Sanger sequenced or cloned into the pGEM T-Easy vector (Promega, Road Madison, WI, USA), and plasmid DNAs from selected clones were sequenced using vector-specific primers (Eurofins Genomics, Ebersberg, Germany).

### 2.3. RNA Extraction and cDNA Synthesis

Total RNA was extracted from 0.05 g of fresh leaf blades using either the GeneJET Plant RNA Purification Kit (Thermo Fisher Scientific) or the Ribospin Plant Kit (GeneAll, Seoul, Korea) following the manufacturer’s protocol. Usually, tissues from two leaves per plant were sampled. The quality and quantity of the RNA was checked with NanoDrop 1000 (Thermo Fisher Scientific) and Qubit RNA HS assays (Invitrogen, Carlsbad, CA, USA). Approximately 100 ng of total RNA was used for cDNA synthesis primed with random hexamers in a 10 µL reaction using the M-MLV Reverse Transcriptase Kit (Invitrogen) according to the manufacturer’s recommendations.

### 2.4. Reverse Transcription Polymerase Chain Reaction (RT-PCR) and Sanger Sequencing

Herbaceous experimental host plants, grafted *F. vesca* indicator clones, strawberry plants from different localities and weeds were analyzed for the presence of StrV-1, and some of them were also analysed for SMoV, SCV, SMYEV, SVBV, and SPV-1 via RT-PCR and Sanger sequencing.

All primers used are listed in Table S2. For two-step RT-PCR, 1 µL of cDNA preparation was added to a mixture of 10 µL of 2× PPP Master Mix (Top-Bio, Vestec, Czech Republic), 8 µL PCR-grade H_2_O, and 0.5 µL of each 0.2 µM primer. For PCR detection of StrV-1 with primers 2f/7r, the reaction conditions were 35 cycles of 95 °C for 15 s, 51 °C for 30 s, and 72 °C for 40 s, with initial denaturation at 94 °C for 2 min and a final extension at 72 °C for 10 min. The primers and cycling conditions for SMoV, SCV, SMYEV, SVBV, and SPV-1 were as described by Thompson et al. [31] and Thekke-Veetil and Tzanetakis [16], respectively.

Genomic 5′ and 3′ terminal amplification was performed with 5′ and 3′ RACE Kits (Invitrogen), respectively, and StrV-1-specific primers. For 3′ terminal amplification, a prior polyadenylation of total RNA with polyU polymerase (NEB, Ipswich, MA, USA) was performed following the manufacturer’s recommendations.

The PCR products thus obtained were resolved on 1% agarose gels, stained with GelRed (Biotium, Hayward, CA, USA), and photographed under UV illumination. For Sanger sequencing, the bands were excised from the gel and purified using the GenElute Gel Extraction Kit (Sigma-Aldrich, Steinheim, Germany). The purified PCR products were sequenced from both directions (Eurofins Genomics).

### 2.5. Two-Step Reverse Transcription Quantitative Polymerase Chain Reaction (RT-qPCR)

For the detection of StrV-1 ČRM1 A, B, and C genotypes, two-step RT-qPCR was performed using cDNA synthesized from 500 ng of total RNA as mentioned above. One microlitre of cDNA was mixed with 0.25 µL of 10 µM primer mixture (final concentration 250 nM), 2 µL of 5× HOT FIREPol^®^ EvaGreen^®^ qPCR Mix Plus (no ROX) (Solis BioDyne, Tartu, Estonia) and supplemented with molecular grade water to a total volume of 10 µL. As an endogenous control, AtropaNad2.1a and AtropaNad2.2b primers were used [31]. Each reaction was run in duplicate.

The efficiency of the qPCR assays was estimated using five 1:10 serial dilutions of in vitro synthesized viral RNA templates in total RNA from a StrV-1-negative sample. For each StrV-1 genotype, individual RNAs were produced with the TranscriptAid T7 High Yield Transcription Kit (Thermo Fisher Scientific) using pGEM T-Easy pDNA templates with 1589:1588 inserts (portions of the P gene from each StrV-1 genotype). The reactions were run using Bio-Rad CFX96 (Bio-Rad, Hercules, CA, USA), and the results were processed using Bio-Rad CFX manager v. 3.1. The efficiency values of the primer pairs are listed in Table S2.

To estimate the genotype abundance of StrV-1 genomic RNAs, RT-qPCR was performed using cDNA synthesized with a mixture of specific primers 1535 (the P gene of SCV) and 1589 (the P gene of StrV-1) to synthesize cDNA from the genomic RNA strand. Each reaction was run in triplicate. SCV was used as the reference to calculate the relative levels of StrV-1 genotypes within each sample.

### 2.6. HTS

Sequencing libraries from total RNA with preceding RiboZERO (Illumina, San Diego, CA, USA) treatment were prepared using either the MuSeek Library Preparation Kit, Illumina compatible (Thermo Scientific, Lithuania) as described earlier [32] or the NEBNext Ultra II Directional RNA Library Prep Kit for Illumina (NEB) and then processed on a HiSeq 4000 platform in 100 b SE output mode (SEQme s.r.o., Dobris, Czech Republic). For amplicon sequencing, PCR products obtained with Q5^®^ High-Fidelity 2× Master Mix (NEB) and StrV-1-generic primers were fragmented, adapter-ligated, and then processed using the Ion Proton system (SEQme, Dobris, Czech Republic).

### 2.7. Sequence and Data Analyses

Sequence analyses were performed with Vector NTI 8 (Invitrogen), Geneious 9.1.5 (Biomatters, Auckland, New Zealand), and CLC Genomics Workbench 8.5.1 (Qiagen, Hilden, Germany). Nucleotide sequences and in silico translated sequences were compared using BLAST+ [33] against GenBank (April 2019) and custom local databases. Phylogenetic analyses were performed using the Phylogeny.fr service [34], and the phylogenetic trees thus obtained were visualized using the iTOL v3 tool [35]. Predictions of functional domains were performed with the Conserved Domain Database v. 3.17 [36], and transmembrane regions were predicted with TMHMM Server v.2.0 [37]. Statistical analysis was performed using RStudio 1.1.463 (R version 3.5.1).

### 2.8. Aphid Transmission of Strawberry Viruses to Herbaceous Hosts and F. vesca ‘Alpine’ Plants

All experiments with aphids were conducted in custom-built mesh cages under temperature-controlled conditions at 21 °C.

Approximately 25 *Aphis fabae* (Scopoli, 1763) individuals were transmitted from naturally infected, symptomatic *F. vesca* (the 1/2017 isolate) to each of eight *Nicotiana occidentalis* 37B plants on August 8, 2017.

*Acyrthosiphon malvae* (Mosley, 1841) (only two individuals were available) was found naturally feeding in gardens on *F. vesca*. Aphids were transferred to one plant of *F. vesca* ‘Alpine’ on May 10, 2019.

Approximately 25 individuals of *Aphis ruborum* (Börner & Schilder, 1931) feeding on flowers and runners of one of the daughter plants of *F. ananassa* ČRM1 were transferred to individual plants of *N. benthamiana* DCL2/4i (*n =* 4), *N. glutinosa* (*n =* 2), *N. occidentalis* 37B (*n =* 8), *N. tabacum* cv. Xanthi (*n =* 8), and *Chenopodium quinoa* (*n =* 7) during April and May 2018. Further transmission experiments were performed by transferring *A. ruborum* from *F. ananassa* (41/2016 and 24/2016 isolates, both plants tested positive for StrV-1 via RT-PCR) to *N. occidentalis* 37B (*n =* 3) and *F. vesca* ‘Alpine’ (*n =* 3) plants in May 2018, respectively. Additionally, *A. ruborum* was used for virus transmission from *F. ananassa* (the 19/2016 isolate, tested positive for StrV-1 and SMoV via RT-PCR) to *F. vesca* ‘Alpine’ (*n* = 2).

*Aulacorthum solani* (Kaltenbach, 1843), observed feeding on a daughter plant of *F. ananassa* ČRM1 growing in the experimental field in IPMB, was transferred to *F. vesca* ‘Alpine’ (*n =* 7) and *N. benthamiana* DCL2/4i (*n =* 5) on May 17, 2019.

*Chaetosiphon fragaefolii* (Cockerell, 1901), found feeding on the *F. vesca* cv. Rujana 3/2017 isolate (SCV and SMoV positive), were transferred to four *F. vesca* ‘Alpine’ on May 17, 2019.

Virus-free *Myzus persicae* (Sulzer, 1776) was cultivated on seed-grown plants of *Sinapis alba* L. Subsequently, aphids were transferred to and cultivated on *Physalis floridana* previously infected with single StrV-1 genotype B (hereafter StrV-1-1/2017(B)). After two months, approximately 25 aphid individuals were transferred to seedlings of seed-grown *F. vesca* ‘Alpine’ (*n =* 5), *N. occidentalis* 37B (*n =* 8), and *N. benthamiana* DCL2/4i (*n =* 4).

Because *A. fabae* and *A. ruborum* died 48 h post-transmission to herbaceous hosts, the plants were not sprayed with an insecticide. However, *A. malvae, A. ruborum, A. solani, C. fragaefolii, and M. persicae* aphids multiplied on *F. vesca* ‘Alpine’ plants, and *M. persicae* also multiplied on *N. benthamiana* DCL2/4i and *N. occidentalis* 37B plants. Therefore, these plants were sprayed with FAST M (active substance: deltamethrin 0.12 g/L) after either 10 days (*N. benthamiana* DCL2/4i, *N. occidentalis* 37B) or 40 days (*F. vesca* ‘Alpine´) from the beginning of aphids feeding on these hosts. The plants were evaluated daily for symptom development over the course of one month or more, if possible.

### 2.9. Mechanical Inoculation of Herbaceous Host Plants

Fourteen days after the first symptoms were observed on *N. occidentalis* 37B plants infested with *A. fabae*, an inoculum mixture was prepared by homogenizing symptomatic leaves (plant no. 3) in 0.1 mol/L sodium phosphate buffer, pH 7.0, in a 1:5 ratio (*w/v*) with carborundum powder as an abrasive agent. Two or three of the first leaves of differential host plants were gently rubbed with the sap homogenate using a glass pestle. Two plants of each herbaceous host inoculated solely with the buffer and carborundum served as negative (healthy) controls. Host plants were washed 2 h after inoculation and then maintained in insect-proof greenhouses. Symptoms were evaluated daily after inoculation over the course of five months.

The inoculum was used for the mechanical inoculation of 20 accessions of *Cucumis sativus* L., *C. quinoa* Willd., *N. benthamiana* Domin., *N. benthamiana* DCL2/4i, *N. glutinosa* L., *N. occidentalis* Wheeler, accession 37B, *N. occidentalis* Wheeler, accession 67A, *N. rustica* L., *N. tabacum* L. cv. Samsun, *N. tabacum* L. cv. Xanthi, *Phaseolus vulgaris* L. cv. Saxa, *P. floridana* Rybd., and *Pisum sativum* L. cv. Zázrak z Kelvedonu plants. Seeds of the transgenic *N. benthamiana* DCL2/4i plants were kindly provided by Dr. Kriton Kalantidis (Institute of Molecular Biology and Biotechnology, Heraklion, Greece) [38]. The DCL2/4i knockdown plants were used as a host with potentially higher susceptibility to viral infection.

### 2.10. Grafting Transmission

Leaves from *F. vesca* ‘Alpine’ seedlings experimentally inoculated using *A. ruborum* with a mixture of StrV-1 genotypes A, B, and C (the 24/2016 isolate) were grafted onto indicator clones (*n* = 8, each) of *F. vesca* ‘Alpine´, EMC, EMK, FV-72 and UC-6 as previously described [39]. Three of the *F. vesca* ‘Alpine’ plants and one of the *F. vesca* EMC, EMK, FV-72 and UC-6 plants grafted from corresponding healthy indicator plants were included as negative controls. Three grafts were used per grafted plant. Grafted plants were cultivated in insect-proof greenhouses under temperature-controlled conditions at 22 °C. The symptoms were observed from three weeks to three months (EMC, EMK, FV-72, UC-6) or later (Alpine) after grafting. The presence of StrV-1 was detected by RT-PCR, and further identification of StrV-1 genotypes was performed by RT-qPCR.

### 2.11. Transmission Electron Microscopy (TEM)

Ultrathin sections were prepared from the symptomatic leaves of *N. occidentalis* 37B and *N. benthamiana* plants mechanically inoculated with StrV-1 as described earlier [40]. Leaf extracts from the abovementioned plants and from multiplied calyx leaves of graft-inoculated *F. vesca* ‘Alpine’ were negatively stained with 2% uranyl acetate and examined under a JEM 1010 transmission electron microscope.

## 3. Results

### 3.1. Symptoms on Strawberry Plants

**Figure 1:**
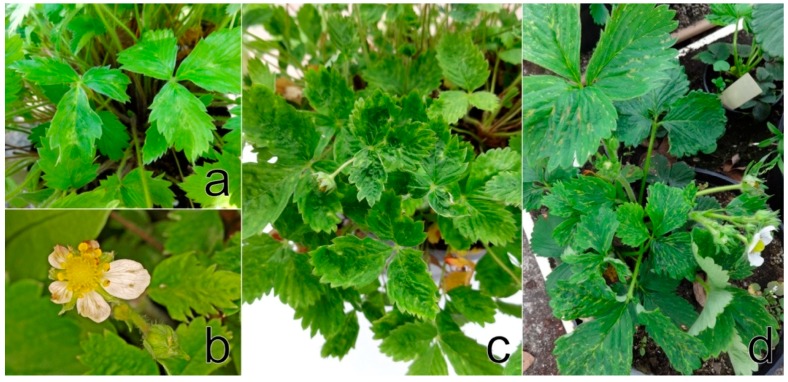
Virus-like symptoms on naturally infected strawberry plants *Fragaria vesca* var. *semperflorens* cv. Rujana (the 1/2017 isolate) showing (**a**) light-green circles, streaks, and blotches in the early stage of infection in June 2016, (**b**) malformed flower with petals unusual in size and shape and petal streaks, (**c**) chlorotic spots and leaflets with the area on either side of the main vein differing in size and shape (May 2017), and (**d**) *F. ananassa* x Duch. Cv. Elkat (the 9/2018 isolate) showing leaf twisting, severe irregular vein clearing and necrosis (May 2019).

*F. ananassa* cv. Čačanská raná plants showed a narrowing of each leaflets, severe leaf malformation, irregularly sized and shaped flower petals and a reduced number of pollen stamens in flowers, as previously demonstrated by Koloniuk et al. [28]. *F. vesca* cv. Rujana exhibited light-green circles, streaks, and blotches on leaves and red-brown lesions and premature redness on the edges of older leaves during the first year of cultivation in 2016. The manifestation of symptoms continued with growth abnormalities, such as dwarfism, leaf and flower deformation, leaf mosaic, and premature leaf drying. Leaflets were uneven in size, distorted and crinkled. Flower petals were unusual in size and shape, and petal streaks were observed on some flowers. Fruits were deformed and reduced in size and numbers (Figure 1a–c). The wild-growing *F. vesca* plants examined showed yellow rings, patterns and spots similar to those observed on Rujana at the early stage of infection. The majority of *F. ananassa* plants screened for StrV-1 revealed non-specific symptoms such as dwarfism and preliminary reddening. The plants of cv. Elkat often showed severe irregular vein clearing and necrosis, regardless of the locality of cultivation (Table S1). Approximately 20% of plants with these symptoms were observed in one production field in South Bohemia (Figure 1d).

### 3.2. HTS

Approximately 100, 50, and 35 million reads were obtained via HTS of samples from *F. ananassa* ČRM1, *F. vesca* cv. Rujana 1/2017, and *F. ananassa* cv. Elkat 9/2018, respectively.

The obtained data for *F. ananassa* ČRM1 were described previously, including the data analyses [28]. The list of viral hits included SCV, SmoV, and a number of entries distantly related to tomato yellow mottle-associated virus that presumably belonged to a novel virus, tentatively named strawberry virus 1. Subsequent analyses indicated the presence of several isolates (genotypes) of all three listed viruses within the analyzed sample. Two complete genomes of SCV have previously been described [28], and six complete protein-coding sequences of SmoV, three for each genomic segment, were deposited in GenBank under accession numbers MH0133322-7. For StrV-1-ČRM1, complete genomic sequences were obtained as described below.

An *F. vesca* cv. Rujana plant, the isolate 1/2017, hosted six different viruses: SCV (two genotypes) and StrV-1 (two genotypes), SPV-1, SmoV, olive latent virus 1, and a novel virus related to viruses of the genus *Umbravirus*. One viral agent, genotype B of StrV-1, was found in the 9/2018 isolate of *F. ananassa* cv. Elkat.

### 3.3. Completion the Genomic Sequence of StrV-1 Genotypes and Their Abundance in the ČRM1 Isolate of F. ananassa

**Figure 2:**
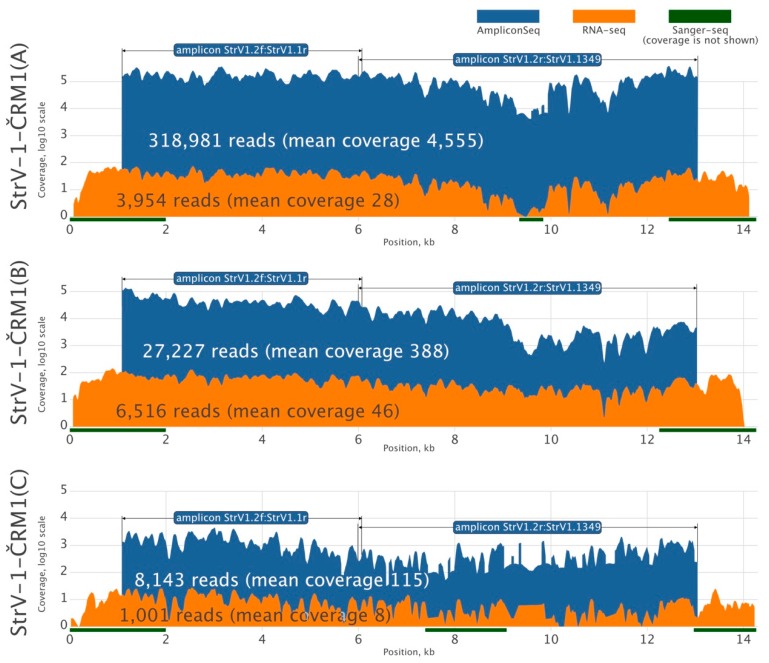
Overview of high-throughput sequencing of genomic A, B, and C genotypes of the isolate Čačanská raná M1 of strawberry virus 1 (StrV-1-ČRM1). Sequence coverages obtained after RNA-Seq and Ion Proton amplicon sequencing are shown in dark orange and blue, respectively. Sanger-sequenced regions are shown as green lines.

Because there was insufficient RNA-seq data for reliable de novo assembly of all StrV-1-ČRM1 genotypes, we amplified their two overlapping genomic segments using genotype-generic primers and subjected the PCR products to HTS using the Ion Proton platform with 200 bp long reads (Figure 2). Three assembled StrV-1 sequences were arbitrarily named genotypes A, B, and C.

These data were supplemented with Sanger sequencing of 3′ and 5′ RACE products to obtain 14,162, 14,028, and 14,255 nucleotide (nt) long complete sequences of all three StrV-1-ČRM1 isolates, A, B, and C, respectively (GenBank accessions MK211270-2).

**Figure 3:**
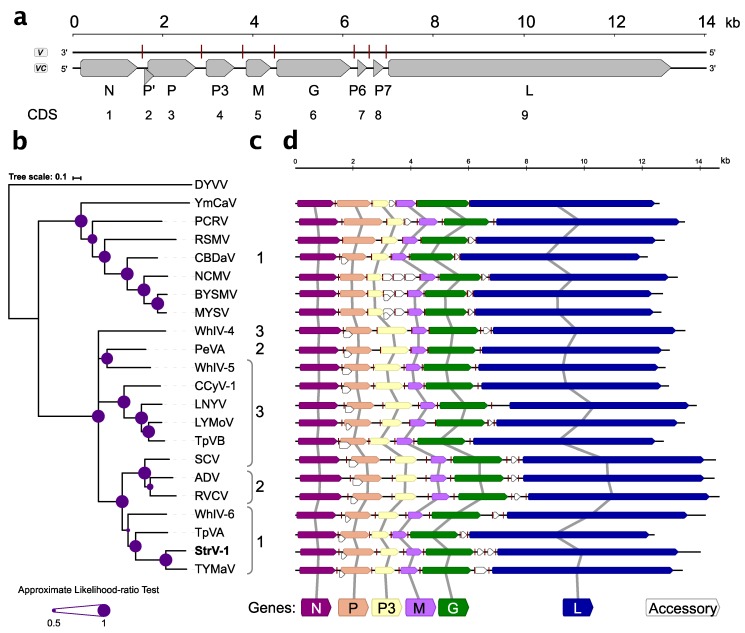
(**a**) Genomic organization of StrV-1-ČRM1, genotype A (MK211270). The nucleotide (nt) sequence is shown as a black line. Viral genomes and complementary strands are labelled as *v* and *vc*, respectively. Open reading frames (ORFs, or CDSs) are drawn as grey arrows, and putative regulatory regions are shown as red vertical lines. Segments are drawn to scale; (**b**) Phylogenetic tree based on L protein sequences of StrV-1 ČRM1 (B) and related cytorhabdoviruses. The acronyms are used as follows: alfalfa dwarf virus, ADV (NC_028237); barley yellow striate mosaic cytorhabdovirus, BYSMV (NC_028244); cabbage cytorhabdovirus 1, CCyV-1 (KY810772); colocasia bobone disease-associated virus, CBDaV (NC_034551); datura yellow vein nucleorhabdovirus, DYVV (NC_028231); lettuce necrotic yellows virus, LNYV (NC_007642); lettuce yellow mottle virus, LYMoV (NC_011532); maize yellow striate virus, MYSV (KY884303); northern cereal mosaic cytorhabdovirus, NCMV (NC_002251); papaya cytorhabdovirus, PCRV (KY366322); persimmon virus A, PeVA (NC_018381); raspberry vein chlorosis virus, RVCV (MK257717); rice stripe mosaic virus, RSMV (NC_040786); strawberry crinkle cytorhabdovirus, SCV (MH129615); tomato yellow mottle-associated virus, TYMaV (NC_034240); Trifolium pratense virus A, TpVA (MH982250); Trifolium pratense virus B, TpVB (MH982249); Wuhan Insect virus 4, WhIV-4 (NC_031225); Wuhan Insect virus 5, WhIV-5 (NC_031227); Wuhan Insect virus 6, WhIV-6 (NC_031232); yerba mate chlorosis-associated virus, YmCaV (KY366322). A plant-infecting nucleorhabdovirus, DYVV, was used as an outgroup. Scale bars refer to a phylogenetic distance expressed in amino acid substitutions per site. Support values are expressed graphically; (**c**) The number of predicted transmembrane domains (TM) in P’ proteins (when applicable); (**d**) Comparative genomic organization of StrV-1-ČRM1 (A) and other cytorhabdoviruses. Virus names are those from the phylogenetic tree. Shared gene synteny is shown with grey lines.

Nine open reading frames/coding sequences (ORFs/CDSs) were identified during sequence analysis (Figure 3a). With the exception of overlapping P’/P, all other CDSs were separated by intergenic sequences.

Five canonical CDSs present in all known rhabdoviruses, N, P, M, G, and L, were identified (Figure 3a,d). The genomic organization of StrV-1-ČRM1 resembles that of other related cytorhabdoviruses with the exception of an additional predicted CDS, tentatively named P7 (Figure 3a,d). Thus far, this is the first described cytorhabdovirus with two putative small CDSs between the G and L CDSs. Neither prediction of functional domains with the Conserved Domains search nor BLASTP searches against nonredundant GenBank database returned any significant hits (cut off E-value 10^−3^, July 10, 2019).

The P’ CDS of StrV-1-ČRM1 encodes a small 7.5 kDa protein that has one predicted transmembrane domain. Interestingly, analyses of the P’ proteins of other cytorhabdoviruses showed that they contained one to three transmembrane domains (Figure 3c).

**Figure 4:**
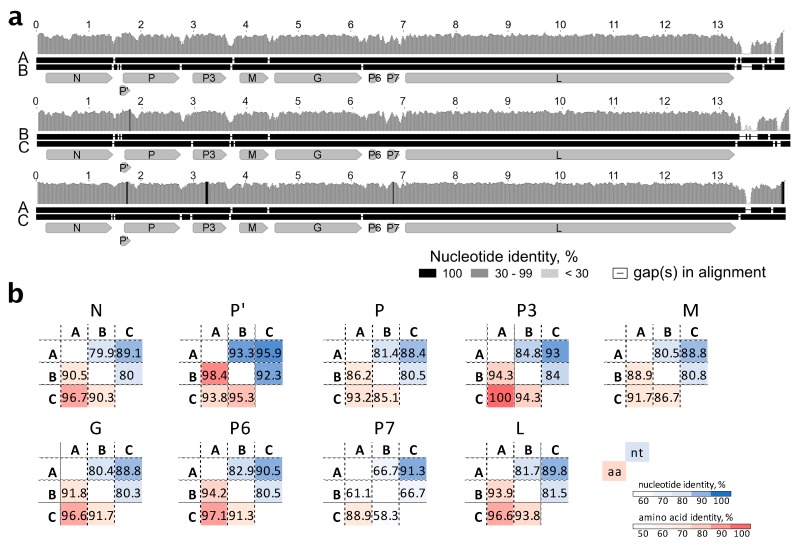
(**a**) Representation of pairwise nt alignments between different genomic genotypes of StrV-1-ČRM1. The *vc* strands were aligned. Gap regions in alignments are shown as lines. A shade legend of nt identities is shown in the lower right corner. Nucleotide positions are shown above each alignment; (**b**) Sequence nt and aa identities between individual genes and putative proteins of three StrV-1-ČRM1 genotypes.

The three genomic genotypes of StrV-1-ČRM1 shared 80% overall nt identity, with the A and C genotypes being less divergent (87% nt identity) and the B genotype showing more differences with A and C, 77% and 76%, respectively (Figure 4a). All insertions/deletions were in the untranslated region of the 5′ terminus. For all CDS, the StrV-1-ČRM1 genotypes A and C shared higher nt and aa identities with each other than with those of the B genotype (Figure 4b).

**Figure 5:**
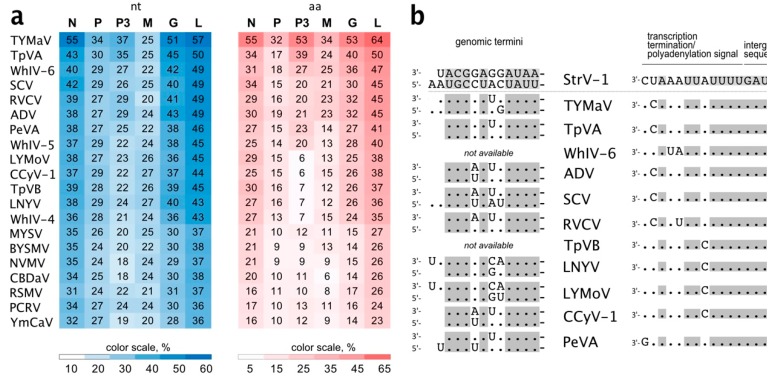
(**a**) Sequence nt and amino acid (aa) identities between genes of StrV-1-ČRM1 (B) and other cytorhabdoviruses; (**b**) Alignment of genomic termini and regulatory sequences of StrV-1-ČRM1 and related cytorhabdoviruses. The accession numbers are the same as those shown in Figure 3.

Notably, StrV-1-ČRM1 isolates shared 53.8%–54.3 % overall nt identity with TYMaV (Figure 5a). Moreover, three CDSs, P, P3, and M, were more divergent, showing from 25% to 37% nt identities (Figure 5a). This is lower than the accepted shared identity between rhabdoviruses of the same species. Twelve nucleotides of the StrV-1-ČRM1 genomic termini were complementary, with 1 nt overhang at the 5′ genomic terminus (Figure 5b). High nt identities were observed between both the genomic termini and regulatory sequences of StrV-1-ČRM1 and related cytorhabdoviruses (Figure 5b).

Phylogenetic analysis based on the L protein sequences placed StrV-1-ČRM1 into one clade with TYMaV, TpVA, and WhIV-6 (Figure 3b). Notably, phylogenetic trees obtained from N, M, and P3 proteins had different topologies (Figure S1).

### 3.4. Ratio of StrV-1 Genotype Abundance in the F. ananassa ČRM1 Sample

**Figure 6:**
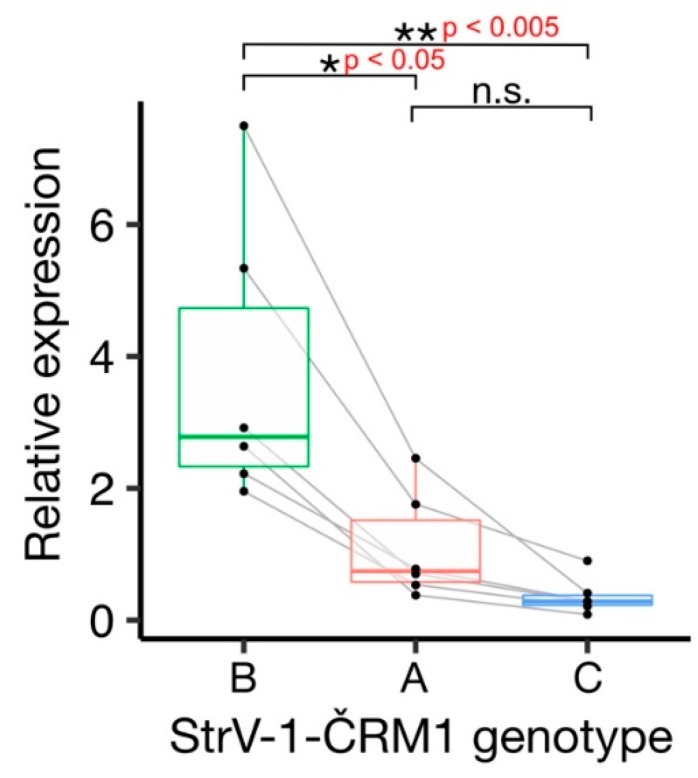
Boxplot graph of the relative abundance of different genotypes of ČRM1 genomic RNAs within individual biological replicates of the ČRM1 isolate of *F. ananassa*. Strawberry crinkle virus was used as a reference. Grey lines connect data points obtained from the same biological replicate.

The affinities of the primers used for amplicon sequencing were not equal, and the StrV-1 genomic genotypes were represented by substantially different numbers of reads (Figure 2). They did not follow the pattern obtained from the RNA-seq data (B > A > C), and for this reason, an RT-qPCR verification was performed. One-way analysis of variance (ANOVA) of the obtained results indicated a significant variation among levels of genomic RNAs of StrV-1 genotypes (F (2,15) = 10.36, *p* < 0.005). A post hoc Tukey test showed that both A:B and B:C pairs differed significantly at *p* < 0.05 and *p* < 0.005, respectively, while differences between A:C were not significant at *p* = 0.62 (Figure 6).

### 3.5. Aphid Screening

The natural presence of *A. fabae* was observed on the lower parts of petioles of *F. vesca* cv. Rujana growing in a private garden in Třísov during the summer of 2016. In April 2017, twelve plants were individually planted in pots and moved to the experimental field at IPMB. Then, aphids were observed on all of these plants and colonized newly growing buds and flowers. Starting in June 2017, strawberry plants growing in the experimental field were sprayed annually with FAST M to eliminate aphid dissemination. Nevertheless, during the following year, the plants were attacked by other aphid species (as described below).

In 2018, one daughter plant of *F. ananassa* ČRM1 and the next few *F. ananassa* plants were naturally infested with *A. ruborum*. Aphids multiplied especially at the tips of newly growing runners, buds and flowers. Colonies of *Macrosiphum euphorbiae* (Thomas, 1878) occupied some strawberry plants and weeds growing among strawberries. During the summer of 2018, *M. persicae* was discovered sporadically on the petioles and young leaves of *F. vesca* cv. Rujana in a gardening shop in České Budějovice.

**Figure 7:**
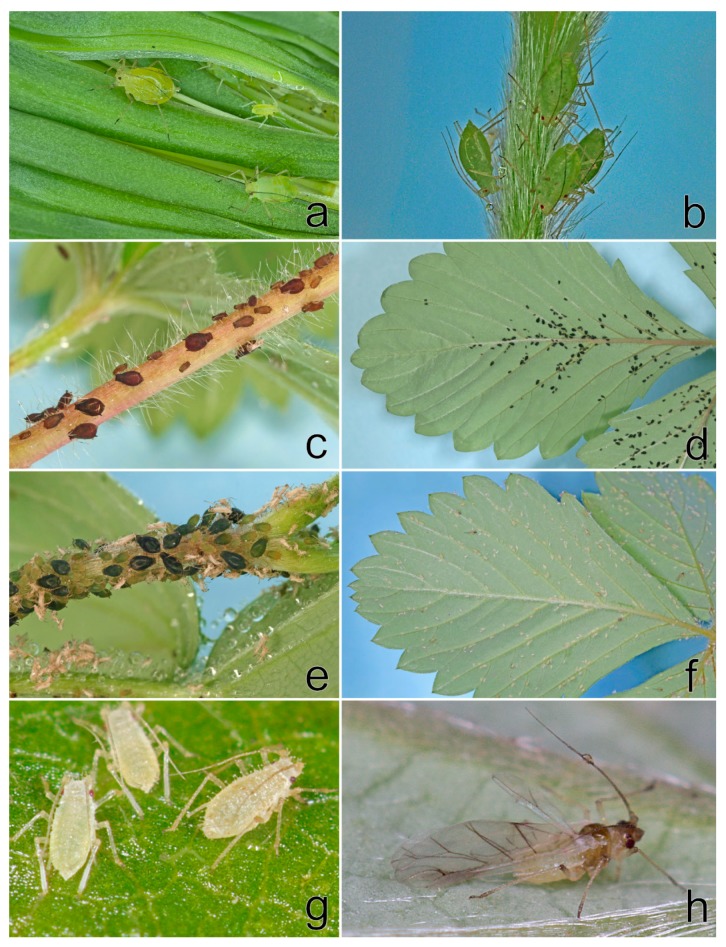
Aphids infesting strawberry plants in South Bohemia in 2019: (**a**) *Aulacorthum solani*, (**b**) *Acyrthosiphon malvae*, (**c**,**d**) *Aphis sanguisorbae*, (**e**) *Aphis forbesi*, (**f**–**h**) *Chaetosiphon fragaefolii*.

In spring 2019, *A. solani* on strawberries and weeds and *C. fragaefolii* feeding on *F. vesca* cv. Rujana in the experimental field were observed. The following aphid species were found infesting strawberries in private gardens in South Bohemia: *A. forbesi* (Weed, 1889) (at newly purchased seedlings of *F. ananassa* potted in autumn 2018: locality Boršov nad Vltavou), *A. malvae* (*F. vesca*: Třísov, Ostrolovský Újezd), *A. sanguisorbae* (*F. vesca*: Třísov, *F. ananassa*: Jamné), and *C. fragaefolii* (*F. ananassa*: Jamné). The aphids detected on strawberries in 2019 are shown in Figure 7.

To confirm the morphological identification of the aphids, PCR was conducted to amplify partial sequences of the *COI* and *cytb* genes. Nucleotide sequences were deposited in the GenBank database (Table 1).

### 3.6. Transmission of Strawberry Viruses to Experimental Host Plants

The mechanical transmission of StrV-1 using crude sap inoculation of strawberry tissues homogenized in different buffers to hundreds of *N. occidentalis* 37B and *C. quinoa* plants repeatedly resulted in negative results in our hands 

**Figure 8:**
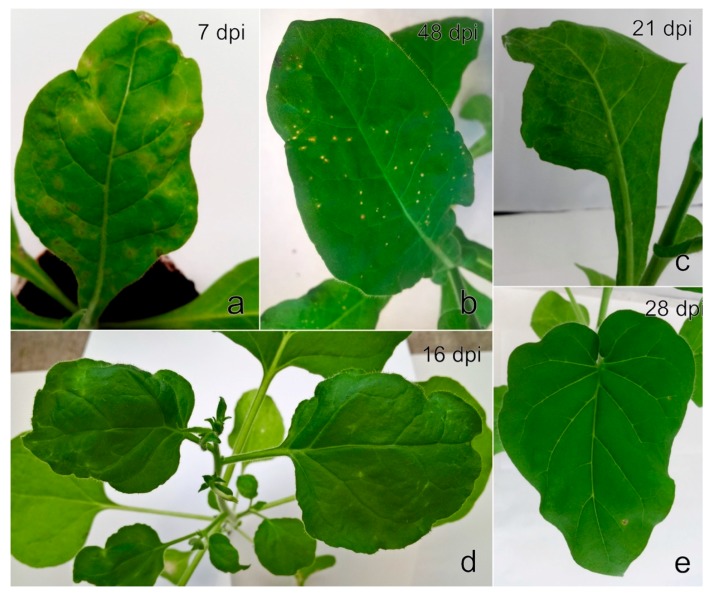
*Nicotiana occidentalis* 37B infected with StrV-1-1/2017 genotype B via *A. fabae* showing (**a**) yellowing and chlorotic and necrotic lesions one week from the beginning of aphid sucking and (**b**) systemic necrotic spots on identical plants in later stages of infection. (**c**) *N. occidentalis* 37B, (**d**) *N. benthamiana* DCL2/4i, and (**e**) *N. glutinosa* infected simultaneously with StrV-1-ČRM1 genotypes A and B by *A. ruborum* feeding exhibited light-green mosaic, irregular vein clearing with chlorotic dots, and sporadic necrotic lesions, respectively.

Five days after the feeding of *A. fabae* on *N. occidentalis* 37B seedlings, mild systemic chlorosis, mosaic, and necrosis were observed on seven out of eight plants examined. Symptoms were more pronounced during the next two weeks (Figure 8a); subsequently, the plants revealed systemic necrotic spots (Figure 8b). Subsequent RT-PCR and Sanger sequencing revealed the presence of a single StrV-1 genotype (StrV-1-1/2017(B)) in all seven symptomatic *N. occidentalis* 37B plants, with the exception of two plants, in which co-infection with SMoV was detected.

**Figure 9:**
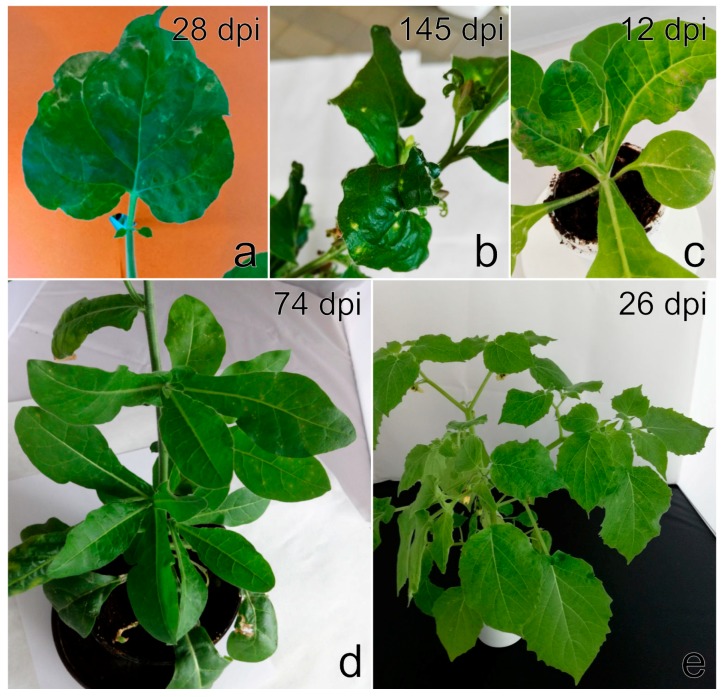
Symptoms on host plants mechanically inoculated with the 1/2017 isolate of StrV-1 (genotype B). (**a**) *N. benthamiana* leaf with mosaic and severe white sectors and (**b**) symptom development to systemic leaf twisting with irregular white lesions; (**c**) *N. occidentalis* 37B showing severe blistered mosaic, yellowing and necrosis at the initial phase of infection and (**d**) identical plants in the later stage of infection, which had systemic necrotic spots; (**e**) *Physalis floridana* showing systemic mild mosaic.

The following mechanical inoculation of differential host plants caused slight to severe mosaic on two of each *N. benthamiana*, *N. benthamiana* DCL2/4i, and *N. occidentalis* 37B plants, and on one *P. floridana* plant at an early stage of infection (Figure 9a,c,e).

A few months later, the *N. benthamiana* plants revealed systemic leaf deformation, white streaks and lesions (Figure 9b), and the *N. occidentalis* 37B plants produced mild systemic mosaic with necrotic spots (Figure 9d). The symptoms on infected *P. floridana* plants were less pronounced, and the plants showed mild dwarfism and smaller leaves in comparison to the control plants. No obvious symptoms were observed on *N. benthamiana* DCL2/4i. RT-PCR and Sanger sequencing revealed the presence of a single genotype, StrV-1 (B), in all symptomatic herbaceous hosts. Further mechanical inoculation of any other herbaceous host plants failed.

The StrV-1-1/2017(B) isolate is maintained to date on cuttings of *P. floridana* in a greenhouse as recently described [41]. The freeze-dried leaves were deposited in UPOC collection funded by the Ministry of Agriculture of the Czech Republic as a part of the National Program of Genepool Conservation of Microorganisms and Small Animals of Economic Importance (https://www.vurv.cz/collections/vurv.exe/search) under accession number UPOC-VIR-044.

*A. ruborum* transmitted different genotypes of StrV-1 individually or in combinations (as later recognized by Sanger sequencing and RT-qPCR, Table S3) from *F. ananassa* ČRM1 to *N. occidentalis* 37B (seven out of eight plants), *N. benthamiana* DCL2/4i (three out of four plants) and *N. glutinosa* (one of two plants). *A. ruborum* further transmitted genotype StrV-1-41/2016(C) from *F. ananassa* 41/2016 to one of three *N. occidentalis* 37B plants; genotypes A, B, and C of StrV-1-24/2016 from *F. ananassa* 24/2016 to *F. vesca* ‘Alpine’ (A, B, and C genotypes simultaneously to all three plants examined); and A and C genotypes StrV-1-19/2016 and SMoV from *F. ananassa* 19/2016 to one ‘Alpine’ plant. Symptoms observed on *N. occidentalis* 37B and *N. benthamiana* DCL2/4i resembled those caused by the StrV-1-1/2017(B) isolate (Figure 8 and Figure 9), while *N. glutinosa* revealed small systemic necrotic lesions (Figure 8e). No symptoms were observed on *N. tabacum* cv. Xanthi and *C. quinoa* (both RT-PCR negative) or *F. vesca* ‘Alpine’ (RT-PCR positive for StrV-1 alone or in co-infection with SMoV).

**Figure 10:**
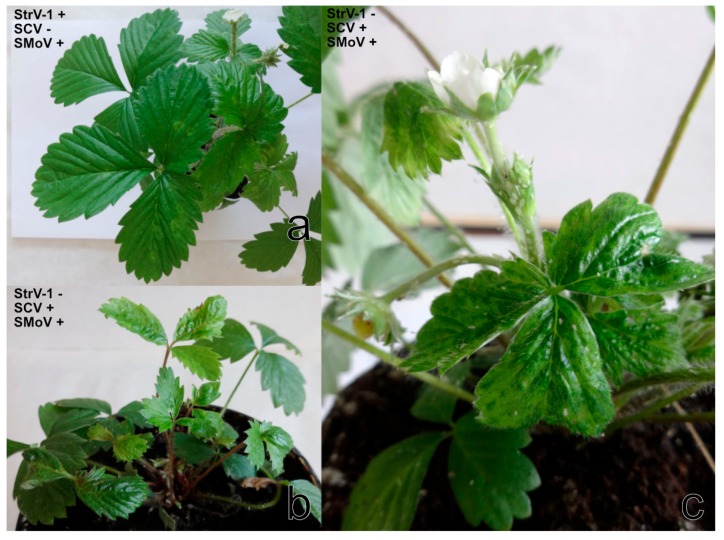
*F. vesca* ‘Alpine’ after virus transmission by aphid feeding: (**a**) *A. malvae* revealed chlorotic markings (RT-PCR positive for StrV-1 and SMoV); (**b**) *A. solani* showed severe dwarfism, leaf malformations, chlorosis and necrosis (SCV and SmoV positive); (**c**) *C. fragaefolii* exhibited severe mosaic and epinasty (SmoV and SCV positive).

Although only two individuals of *A. malvae* were transferred from cultivated *F. vesca* (unknown cultivar) to one *F. vesca* ‘Alpine´, this plant revealed light-green rings, irregular vein clearing and newly growing leaves that curled down (Figure 10a). StrV-1 in co-infection with SMoV was identified in this plant.

*A. solani* did not transmit any virus from *F. ananassa* ČRM1 to *N. benthamiana* DCL2/4i. However, one of seven *F. vesca* ‘Alpine’ plants showed a severe reduction in the size of petioles and leaves, leaflet narrowing, malformation and necrosis (Figure 10b). RT-PCR and Sanger sequencing detected the presence of SCV together with SMoV in this plant.

*C. fragaefolii* transmitted SCV and SMoV (the 3/2017 isolate) to all four examined *F. vesca* ‘Alpine’ plants. The symptoms of severe mosaic and distortion appeared on the youngest leaves starting on the 14th day after feeding (Figure 10c).

When using *M. persicae*, no virus disease-like symptoms were observed either on *N. occidentalis* 37B or on *F. vesca* ‘Alpine’ and *N. benthamiana* DCL2/4i. The presence of StrV-1 or the other abovementioned viruses was not detected by RT-PCR.

All aphid species that were found infesting strawberries in the present work, including those identified by biological assay as potential SCV, StrV-1, and SMoV vectors, are summarised in Table 1.

According to the RT-PCR results, SMYEV, SPV-1, and SVBV were not identified in either the aphid- or mechanically inoculated herbaceous host or *F. vesca* ‘Alpine´plants.

### 3.7. Leaflet Grafting

**Figure 11:**
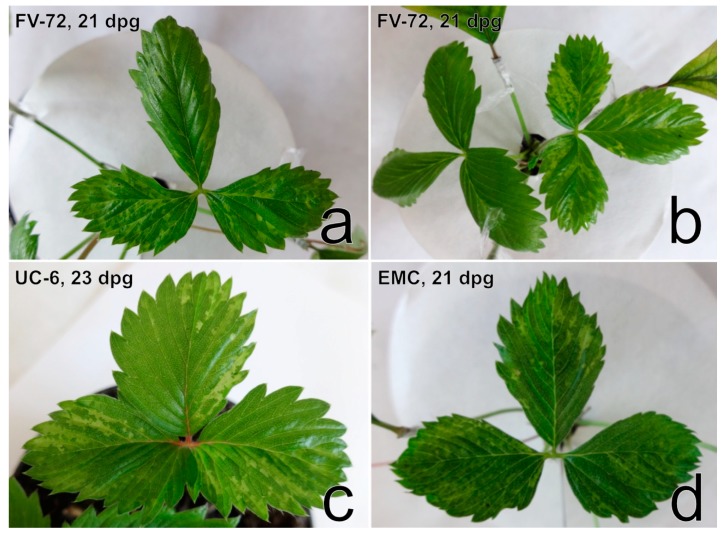
Symptoms of irregular light-green sectors on *F. vesca* clones after grafting. Dpg—days post-grafting.

Manifestations of virus disease symptoms on the four *F. vesca* indicator clonal lines (EMC, EMK, FV-72, UC-6) were similar and pronounced mostly between the 3rd and 4th week after grafting (Table S4, Figure 11).

**Figure 12:**
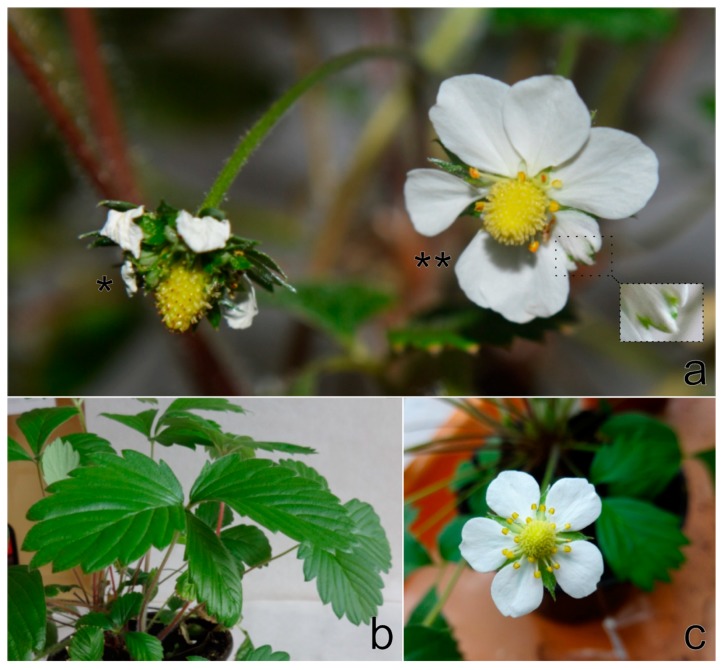
*F. vesca semperflorens* ‘Alpine’ inoculated by grafting with StrV-1-24/2016(A, B, C), exhibiting (**a**) multiplication of calyx leaves on one flower (*), petal deformation together with light-green streaks and an irregular length and number of flower structures on the second flower (**), and (**b**) leaf epinasty 84 days after grafting; (**c**) A flower from the healthy control.

The symptoms involved light-green sectors and/or irregular vein clearing on newly developed leaves and were most noticeable on *F. vesca* plants of the FV-72 line. In the course of the following month, symptoms were less obvious or even barely discernible. The expression of virus disease symptoms on *F. vesca* ‘Alpine’ was less frequent than that on the abovementioned clones (Table S4). Only one ‘Alpine’ plant revealed distortion of leaves with mild irregular vein clearing three weeks after grafting. Two months later, the plant developed epinasty on one leaf a produced only two flowers. One flower showed multiplication of calyx leaves, and the other revealed petal malformations (Figure 12).

However, StrV-1 was RT-PCR identified in 15 symptomatic and nine asymptomatic plants out of 40 grafted plants. The transmission of different StrV-1 genotypes was revealed by RT-qPCR. All three genotypes (StrV-1-24/2016(A, B, C)) were determined in 20 plants; the simultaneous presence of two genotypes (A, C) and only the B genotype was determined each in two plants (Table S4). All grafted healthy controls were RT-PCR negative, although mild chlorotic spots were observed on EMK and EMC three weeks after grafting.

### 3.8. RT-PCR Screening for StrV-1 and Sanger Sequencing

The RT-PCR assay using the 2f/7r primers revealed amplicons of the expected size (327 bp) in symptomatic herbaceous host plants and *F. vesca* indicator clones, as mentioned above. Moreover, StrV-1 was detected in 49 out of 159 (31%) strawberry plants cultivated in the production fields (*n* = 32) and gardens (*n* = 13) in West, East, and South Bohemia, and in South Moravia and in wild growing *F. vesca* plants (*n* = 4) in South Bohemia (Table S1). The virus was not detected in any weeds growing among the cultivated strawberries (*Betula pendula* Roth (*n* = 1), *Carex* sp. (*n* = 1), *Epilobium parviflorum* Schreb. (*n* = 1), *Hypochaeris radicata* L. (*n* = 1), *Poa annua* L. (*n* = 1), *Salix caprea* L. (*n* = 1), *Scorzoneroides autumnalis* (L.) Moench. (*n* = 1), *Sonchus arvensis* L. (*n* = 4), *Stellaria media* (L.) Vill. (*n* = 5), *Taraxacum officinale* Web. (*n* = 5)), although polyphagous aphids (*A. solani*, *M. euphorbiae*) were found infesting strawberries and these weed plants.

However, some chromatograms from Sanger sequencing showed multiple secondary peaks in the examined samples. Comparison with data obtained by HTS and RT-qPCR revealed either the presence of a single StrV-1 genotype or the presence of up to three different genomic genotypes in one host plant. The most frequent was the B genotype of StrV-1 (single or in co-infection with another genotype(s)) (Table S1).

### 3.9. TEM

**Figure 13:**
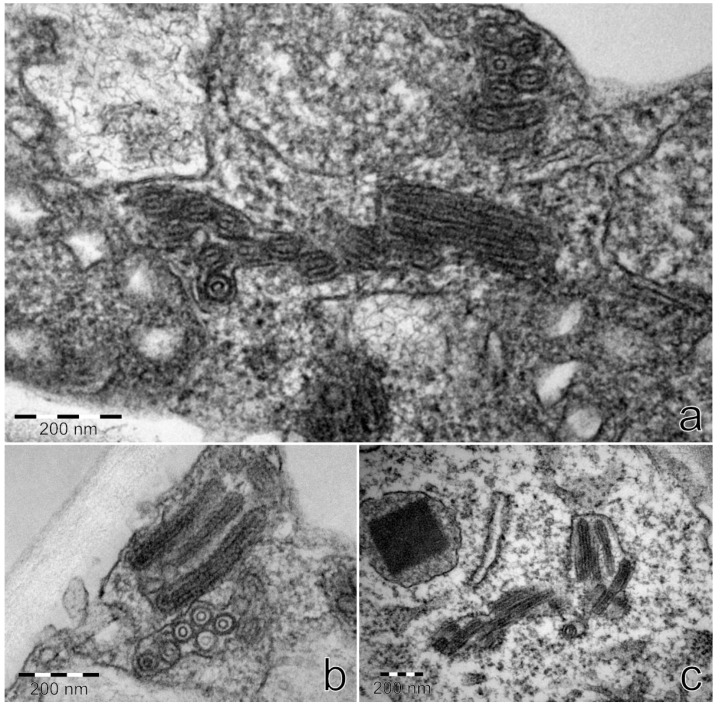
Bacilliform rhabdovirus-like particles in cytoplasmic inclusions on ultrathin sections from *N. benthamiana* (**a**,**b**) and *N. occidentalis* 37B (**c**) plants infected with the StrV-1-1/2017(B) genotype.

Ultrathin sections from StrV-1-1/2017(B)-positive plants *N. occidentalis* 37B and *N. benthamiana* contained bacilliform virus-like particles with rounded ends with dimensions of approximately 53 × 174–285 nm. They were detected in parenchymatous cells and usually surrounded with a membrane (Figure 13). No virus-like particles were found on negatively stained preparations.

## 4. Discussion

Here, we describe a novel cytorhabdovirus infecting strawberry, tentatively named StrV-1. It was found to spread to strawberry production fields and private gardens in *F. ananassa*, *F. vesca* and wild-growing *F. vesca* plants in the Czech Republic. Initial virus discovery was made via the analysis of Illumina HTS data. The complete genomic sequences of three different StrV-1 genotypes with an average of 80% shared nt identities hosted within a single plant of *F. ananassa* cv. Čačanská raná were identified by additional HTS of overlapping amplicons and by performing RACE procedures.

The genomic organization of StrV-1 closely resembled that of TYMaV, except for an additional small CDS P7 positioned before the L CDS. Thus far, this is a unique trait among known cytorhabdoviruses. In addition, nucleotide identity differences between the P, P3, and M genes of StrV-1 and TYMaV were higher than the species demarcation criterion for rhabdoviruses [20,42], which confirmed that StrV-1 and TYMaV were not distant strains of the same cytorhabdoviral species.

During the prediction of TM domains in the StrV-1 putative proteins, an unusual pattern was revealed. There was a single TM region in the P’ proteins of StrV-1, TYMaV, TpVA, and WhIV-6, while other cytorhabdoviruses had from two to three TM domains. The P’ function, however, remains to be uncovered.

Phylogenetic analyses using all protein sequences showed that StrV-1-ČRM1 and TYMaV were consistently placed in the same phylogenetic clade, while TpVA and WhIV-6 were not. This might indicate the occurrence of evolutionary recombination events. However, recombination analyses have not revealed any well-supported recombination events (RDP 3 program cut off, at least five of the used algorithms.

HTS of other strawberry samples revealed the existence of two StrV-1 genotypes in the 1/2017 isolate of *F. vesca* cv. Rujana. Both 1/2017 and ČRM1 plants were co-infected with other viruses. As previously reported for the most important aphid-borne strawberry viruses, especially SMoV and SCV, infections with various virus strains seem to be a common phenomenon in strawberry crops [2,3,9,10,28,43]. Notably, using HTS, we determined a single StrV-1 genotype in *F. ananassa* cv. Elkat, without apparent co-infection with any other viruses. This plant, however, showed symptoms of permanent severe vein clearing and necrosis that were also observed in daughter plants. Surprisingly, the RT-qPCR StrV-1 genotype determination assay and analyses of Sanger chromatograms revealed only the genotype B in all plants of *F. ananassa* cv. Elkat, regardless of the locality of growing. In contrast, wild strawberries as well as garden strawberries were predominantly co-infected with two or three StrV-1 genotypes. This can be caused by the long-term cultivation of different strawberry cultivars of different origins in the same place and/or the presence of insect vector(s) in the gardens. Nevertheless, the determination of StrV-1 genotypes based on RT-PCR and Sanger sequencing was only partially in agreement with the RT-qPCR assays. Thus, we cannot exclude either the existence of other StrV-1 genotypes or substantial nt differences within the RT-qPCR primer binding sites. The described RT-PCR protocol, in which a portion of the N gene was amplified from three revealed StrV-1 genotypes, can be utilized as a tool for StrV-1 detection in field and experimental studies.

It should be noted that during peer review of the current manuscript, a virus named ‘strawberry-associated virus 1′ (GenBank accession MK159261) was reported in the single strawberry plant in China [44]. It shared 98.3% of nt identity with the genotype A of StrV-1-ČRM1. Performed RT-PCR testing of 113 strawberry plants in Fujian province did not reveal the virus presence. However, based on the used primers [44] it is possible that the RT-PCR assay was limited only to the A virus genotype.

The only cytorhabdoviruses described thus far in strawberry are SCV and abovementioned strawberry-associated virus. Rhabdovirus-like particles of 69 ± 6 × 190–380 nm [45] and differently sized bacilliform particles of 74 × 163 nm, 87 × 207 nm and 88 × 383 nm were described for SCV [46]. Twenty years ago, we observed only two bacilliform rhabdovirus-like particles of 45–60 × 285–320 nm on negatively stained partially purified preparation from the *F. vesca* FV-72 clone, which was graft-inoculated from a plant of *F. ananassa* cv. Čačanská raná [47]. Therefore, we suggest that the abovementioned particles could be particles of StrV-1, since measurement of StrV-1-1/2017(B) particles on ultrathin sections revealed shorter and narrower (53 × 174–285 nm) sizes than those previously described for SCV. Moreover, as previously reported [48], the measurement of the size of rhabdovirus-like particles is only approximate because of shrinkage taking place during the dehydration of specimens for electron microscopy.

Crude sap transmission of StrV-1 directly from strawberry to herbaceous hosts failed. The initial transfer of StrV-1 by aphids to *N. occidentalis* 37B was necessary. Subsequent mechanical inoculation allowed us to transmit StrV-1B to *N. occidentalis* 37B, *N. benthamiana*, *N. benthamiana* DCL2/4i and *P. floridana*. Of the known aphid-borne strawberry viruses, SCV was previously mechanically transmitted to *N. occidentalis* subsps. *obliqua*, *N. clevelandii*, and *P. pubescens* after initial transmission by aphids to *N. occidentalis* or *P. pubescens* [46]. SMoV has also been transmitted to plants outside the genus *Fragaria* by mechanical means. In some cases, however, it was necessary to transmit SMoV to *C. quinoa* by means of aphids before it could be further mechanically transmitted [9].

Prior to the development of molecular assays, leaflet grafting was the single most reliable method for detecting strawberry viruses. Its broad detection spectrum is extremely valuable in identifying poorly characterized viruses [3,9]. Therefore, the aim of grafting in this study was to shed light on symptoms caused by StrV-1 on *F. vesca* indicator clones. Based on the observed symptoms, however, it was not possible to conclusively recognize StrV-1 infected plants, since nine out of 40 grafted indicator clones did not reveal any virus disease-like symptoms, although the plants were StrV-1-positive based on RT-PCR assays. Moreover, the symptomatology of *F. vesca* did not permit unequivocal differentiation between StrV-1 and similar symptoms produced by SMoV, SVBV or strawberry chlorotic fleck agent, alone or in combination [3]. In addition, chlorotic spots observed on graft-indexed plants and on *F. vesca* EMK and EMC controls can be considered heat spots and can be results of physiological stress [9]. Only the multiplication of calyx leaves on *F. vesca* ‘Alpine’ seems to be a unique diagnostic symptom of the presence of StrV-1 and has not been previously described for other virus infection. To date, petal streaks have been described as diagnostic symptoms for SCV [3]. To our knowledge, similar symptoms to those observed on StrV-1-infected ‘Alpine’ plants have been previously described as flower phyllody associated with strawberry green petal phytoplasma [40]. It is further characterized by small and red leaves, asymmetrical new leaves and ultimately causing plant death. In our case, the grafted *F. vesca* ‘Alpine’ and donor plant did not show these symptoms characteristic of phytoplasma infection.

*A. fabae*, *A. ruborum*, and *A. malvae* effectively transmitted StrV-1, while *M. persicae* and *A. solani* transmitted StrV-1 to neither the herbaceous hosts nor *F. vesca* ‘Alpine’ in our hands. Additionally, we first report here that *A. fabae* is a vector of SMoV and that *A. solani* is a vector of SCV and SMoV under experimental conditions. We also confirmed the transmission of SMoV by *A. malvae* as well as the transmission of SMoV and SCV by *C. fragaefolii* To our knowledge, *A. solani* has previously been reported to be a vector of SVBV, while *A. fabae* and *A. ruborum* have not been reported as a vector of strawberry viruses until this time [9].

*C. fragaefolii* is distributed worldwide and is the most important vector of viruses in strawberry fields [3]. It is presumed to originate from North America. According to the Centre for Agriculture and Bioscience International, this aphid species was recorded from the southwestern part of Europe, with a widespread distribution in Germany, Bulgaria, and the United Kingdom [49]. The occurrence of *C. fragaefolii* was not reported in the central or northern part of Europe until this time. By morphological and molecular means, we determined the natural occurrence of this important pest in two localities of South Bohemia (Czech Republic). Consequently, this is the first report of the occurrence of *C. fragaefolii* in Central Europe. Most likely, due to climate changes and/or the import of plant material, this species of aphid has expanded from the warmer region to our country. Other polyphagous aphid species, such as *A. fabae*, *A. ruborum*, *A. malvae*, and *A. solani*, may be significant in the spread of strawberry viruses in our climatic conditions. In addition, *A. sanguisorbae* has not been previously found to infest strawberry plants [50].

In future research, the exact transmission of StrV-1 by aphids (non-persistent, semi-persistent, persistent) should be elucidated. Because there is the possibility of specific interactions between pathogens and host genotypes, further research should be conducted to elucidate the sensitivity of different strawberry cultivars to StrV-1 infection and to its different co-infecting genotypes. The distribution of the virus in propagated stock materials and its importance in strawberry production should also be evaluated.

## 5. Conclusions

A novel RNA virus, named strawberry virus 1, infecting *Fragaria ananassa* and *F. vesca* was discovered using HTS. Phylogenetic and sequence analyses indicated that the virus is closely related to members of the *Cytorhabdovirus* genus (*Rhabdoviridae*), which was further confirmed by the morphology of its particles. Successful aphid-mediated and mechanically mediated StrV-1 transmission to experimental plant species was performed. Potential aphid StrV-1 vectors were identified.

For the first time, *C. fragaefolii* was shown to be present in the Czech Republic, and *A. sanguisorbae* was found to infest strawberry plants.

This is also the first report of strawberry polerovirus-1 (*Luteoviridae*) outside of the American continent and of olive latent virus 1 (*Tombusviridae*, *Alphanecrovirus*) infecting strawberries.

## Figures and Tables

**Table 1:** Aphids infesting strawberry plants and experimental host plants of strawberry viruses determined in the present study.

Vector/Acc. Number	Virus	Experimental Host
*Acyrthosiphon malvae/MN420505*	StrV-1, SMoV	*Fragaria vesca* ‘Alpine´
*Aphis fabae* n.d.	StrV-1, SMoV StrV-1	*Nicotiana occidentalis* 37B
		*N. benthamiana*
		*N. benthamiana* DCL2/4i
		*Physalis floridana*
*Aphis sanguisorbae/* *MN420509*	n.d.	n.d.
*Aphis ruborum/* n.d.	StrV-1, SMoV StrV-1	*F. vesca* ‘Alpine´
		*N. occidentalis* 37B
		*N. benthamiana* DCL2/4i
		*N. glutinosa*
*Aulacorthum solani/* *MN420508, MN420511*	SMoV, SCV	*F. vesca* ‘Alpine´
*Chaetosiphon fragaefolii/* *MN420510*	SMoV, SCV	*F. vesca* ‘Alpine´
*Macrosiphum euphorbiae/* n.d.	n.d.	n.d.
*Myzus persicae/* *MN420506*	n.d.	n.d.
*Aphis forbesi/* *MN420507*	n.d.	n.d.

n.d.—not determined.

## Supplementary Materials

The following are available online at https://www.mdpi.com/1999-4915/11/11/982/s1, Table S1: Symptoms and determination of StrV-1 genotypes using Sanger sequencing and RT-qPCR in strawberry plants from different localities of the Czech Republic, Table S2: List of used primers, Table S3: Simultaneous *Aphis ruborum*-mediated transmission of three genotypes of strawberry virus 1 transmitted from *Fragaria ananassa* cv. Čačanská raná to herbaceous host plants, Table S4: Graft-mediated transmission of StrV-1 genotypes to *F. vesca* indicator clones, Figure S1: Phylogenetic trees of N, M, and P3 genes.
